# Aspiration of tissues and ascites: the solution to endoscopic ultrasound-related duodenal perforation during pancreatic cancer sampling

**DOI:** 10.1055/a-2208-5363

**Published:** 2023-12-05

**Authors:** Qin Lu, Zhi-qiang Du, Xiang-rong Zhou, Min Yang, Rui Huang, Wei-hui Liu

**Affiliations:** 1 Department of Gastroenterology and Hepatology, Sichuan Provincial People’s Hospital, School of Medicine, University of Electronic Science and Technology of China, Chengdu, China; 2 Department of Gastroenterology, Jianyang People’s Hospital, Jianyang, China

Duodenal perforation is one of the most severe complications of endoscopic ultrasound-guided fine-needle aspiration (EUS-FNA) 1 2 3 4 5 . Here we report on a patient with a large duodenal perforation during EUS-FNA who was successfully treated under endoscopy with purse-string suture, release of abdominal gas, aspiration of tissues and ascites, and sufficient decompression and drainage ( Video 1 ).

Endoscopic ultrasound (EUS)-guided fine-needle aspiration of ascites and tissues in a patient with EUS-related large duodenal perforation.Video 1

An 80-year-old woman was referred to our unit to undergo EUS-FNA for diagnosis of a pancreatic lesion. After a hypoechoic area approximately 2.1×1.7 cm in size was found in the pancreatic head ( Fig. 1 **a** ), a perforation measuring approximately 1.0 cm in diameter was clearly seen at the junction of the descending duodenum ( Fig. 1 **b** ). The procedure was immediately discontinued, and 10 endoclips and a nylon ring were used to create a purse-string suture of the perforation ( Fig. 1 **c** ). Meanwhile, the free intraperitoneal gas was released by a syringe to reduce the abdominal tension. Then, 600 mL of light-yellow liquid was aspirated from the abdominal cavity by a 19 G puncture needle after complete closure of the perforation was confirmed ( Fig. 1 **d** ). Histological tissues were obtained during repeat EUS-FNA and confirmed the lesion to be pancreatic cancer ( Fig. 1 **e** ). Finally, a nasoduodenal tube with multiple lateral foramina was placed for local drainage and decompression. A repeat abdominal computed tomography scan indicated no newly increased gas and fluid in the abdomen ( Fig. 1 **f** ). The patient remained asymptomatic and was discharged 1 week later.

**Fig. 1:**
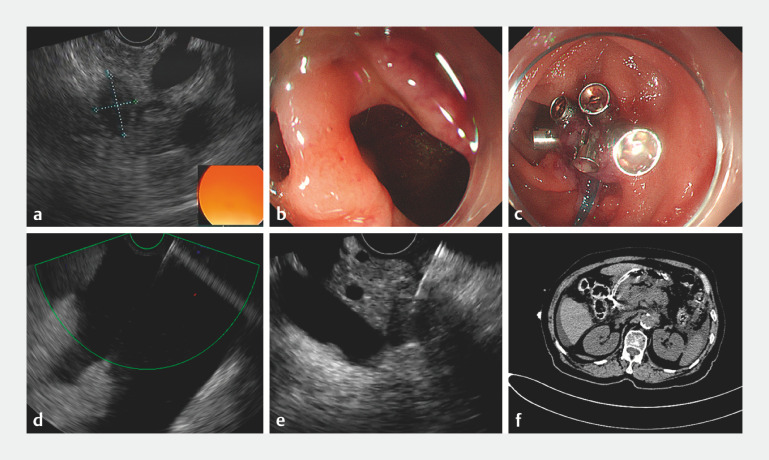
Management of endoscopic ultrasound (EUS)-related duodenal perforation during pancreatic cancer sampling. **a** A pancreatic head lesion was detected on EUS. **b** A perforation measuring approximately 1.0 cm in diameter was seen at the junction of the descending duodenal bulb. **c** Endoscopic image after 10 endoclips and a nylon ring were used to create a purse-string suture of the EUS-related large duodenal perforation. **d** EUS-guided fine-needle aspiration (FNA) was performed to remove large amounts of ascites in the abdominal cavity. **e** Histological tissues were obtained during EUS-FNA. **f** After the endoscopic repair, no new increase in free gas and fluid was detected on computed tomography.

In summary, we used a puncture needle to aspirate both ascites and sufficient tissues after complete closure of the perforation, allowing both clarification of the pathological diagnosis and reduction of the risk of complications, such as infection. This method proved to be beneficial not only for diagnosis but also for closure of the perforation, making it a potential preferred endoscopic therapeutic option for duodenal perforation in the future.

Endoscopy_UCTN_Code_CPL_1AL_2AB

## Notes

Endoscopy E-Videos https://eref.thieme.de/e-videos
*E-Videos* is an open access online section of the journal *Endoscopy* , reporting on interesting cases and new techniques in gastroenterological endoscopy. All papers include a high-quality video and are published with a Creative Commons CC-BY license. Endoscopy E-Videos qualify for HINARI discounts and waivers and eligibility is automatically checked during the submission process. We grant 100% waivers to articles whose corresponding authors are based in Group A countries and 50% waivers to those who are based in Group B countries as classified by Research4Life (see: https://www.research4life.org/access/eligibility/ ). This section has its own submission website at https://mc.manuscriptcentral.com/e-videos .
